# Potential and pitfalls of measuring circulating anti-nephrin autoantibodies in glomerular diseases

**DOI:** 10.1093/ckj/sfaf100

**Published:** 2025-04-12

**Authors:** Felicitas E Hengel, Tobias B Huber, Nicola M Tomas

**Affiliations:** III. Department of Medicine, University Medical Center Hamburg-Eppendorf, Hamburg, Germany; Hamburg Center for Kidney Health, University Medical Center Hamburg-Eppendorf, Hamburg, Germany; III. Department of Medicine, University Medical Center Hamburg-Eppendorf, Hamburg, Germany; Hamburg Center for Kidney Health, University Medical Center Hamburg-Eppendorf, Hamburg, Germany; III. Department of Medicine, University Medical Center Hamburg-Eppendorf, Hamburg, Germany; Hamburg Center for Kidney Health, University Medical Center Hamburg-Eppendorf, Hamburg, Germany

**Keywords:** autoantibodies, glomerulonephritis, minimal change disease, nephrin, nephrotic syndrome

## Abstract

Recent studies have identified autoantibodies targeting the podocyte protein nephrin in patients with primary podocytopathies such as minimal change disease, primary focal segmental glomerulosclerosis (FSGS), post-transplant recurrent FSGS and childhood idiopathic nephrotic syndrome. These antibodies bind nephrin and directly influence nephrin downstream signaling, with immense effect on the podocytes’ cellular structure and function, substantially changing our understanding of antibody-mediated podocytopathies and disease classification. Their presence correlates with disease activity and holds great potential as a novel biomarker of anti-nephrin-associated podocytopathy. However, the detection of these potentially low-titre autoantibodies has proven challenging. In this review, we highlight and explain distinct detection methodologies with their advantages and disadvantages and discuss the potential of anti-nephrin autoantibodies as a novel biomarker in nephrotic syndrome for diagnosis, prognostication and therapeutic guidance in patients with nephrotic syndrome.

## ANTI-NEPHRIN AUTOANTIBODIES IN GLOMERULAR DISEASES

Autoantibodies against nephrin, a key slit diaphragm protein of podocytes, are a newly identified factor in patients with primary podocytopathies such as minimal change disease (MCD) and primary focal segmental glomerulosclerosis (FSGS), and children with idiopathic nephrotic syndrome (INS) [1–6]. Their prevalence varies depending on the cohort and the applied detection method, ranging from 29% to 44% of patients with MCD, 9% of patients with primary FSGS and 38% to 52% of children with INS [1–4] (Table 1). The prevalence is as high as 69% in MCD and 90% in children with INS when selecting immunosuppression-naïve patients with active nephrotic disease and substantially lower in children with steroid-resistant nephrotic syndrome [2, 4]. Anti-nephrin autoantibody levels strongly correlate with nephrotic disease activity, and targeted immunosuppressive treatment with rituximab to deplete antibody-producing cells in individual patients with relapsing anti-nephrin-associated disease was shown to induce sustained clinical and immunological remission in several cases [2].

**Table 1:** Overview of detection methods and prevalence of circulating anti-nephrin autoantibodies across different studies and cohorts.

Study	Detection method of circulating anti-nephrin autoantibodies	Cohort characteristics	Prevalence of anti-nephrin autoantibodies	Further findings related to anti-nephrin measurement
Watts *et al*., *JASN* 2022 [1]	Immunoprecipitation and signal-enhanced ELISA with lab-produced recombinant human nephrin ectodomain, 6xHIS-tagged, expressed in HEK293	– 41 children and 21 adults with biopsy-proven MCD (NEPTUNE cohort) – 54 PLA2R-MN – 30 healthy controls – Further patient samples for paired serum and biopsy-assessment	– 29% (18/62) in MCD – 2% (1/54) in PLA2R-positive MN	– Shorter relapse-free period in anti-nephrin-positive patients compared with anti-nephrin-negative patients (median time to relapse 6.0 months vs 21.57 months; *P* = .09)
Hengel *et al*., *NEJM* 2024 [2]	Immunoprecipitation with lab-produced recombinant human nephrin ectodomain, 8xHIS- and twinstrep-tagged, or hybrid assay of immunoprecipitation followed by ELISA of eluted recombinant nephrin, expressed in HEK293	– 182 children with INS (NEPHROVIR cohort, cohorts from Bari and Rome) – 357 adults with biopsy-proven glomerular diseases (MCD, pFSGS, MN, IgAN, ANCA, SLE from Hamburg GN Registry and Bari cohort) – 117 healthy controls (50 children, 67 adults)	– 52% (94/182) in INS – 44% (46/105) in MCD – 9% (7/74) in pFSGS – 3% (1/40) in non-primary FSGS – 2% (1/50) in PLA2R-positive MN	– Correlation of anti-nephrin titre with proteinuria (Spearman's r = 0.64) – Increasing prevalence of anti-nephrin depending on disease activity and immunosuppression-naïve sampling
Raglianti *et al*., *KI* 2024 [3]	Standard ELISA using a commercial recombinant protein partially covering the first IgG-like domain of human nephrin (AA 23–92), corresponding to approx. 7% of the ectodomain of human nephrin, no information on expression system available	– 12 children with MCD/FSGS – 13 paediatric SSNS – 76 paediatric controls – 19 adults with MCD/FSGS – 17 adult controls	– 33% (4/12) in children with MCD/FSGS – 38% (5/13) SSNS – 11% (2/19) adults with MCD/FSGS – 0% (0/93) controls	– In parallel, kidney biopsies of patients were analysed by super resolution microscopy for IgG colocalizing with nephrin, differentiating into histological anti-slit and anti-nephrin antibody positivity
Hengel *et al*., *KI* 2025 [4]	Immunoprecipitation with lab-produced recombinant human nephrin ectodomain, 8xHIS- and twinstrep-tagged or 8xHIS-tagged	– 333 children with NS (NEPHROVIR cohort, PODONET cohort, cohort from Rome): – 101 SSNS – 67 SDNS – 103 non-genetic SRNS – 62 genetic SRNS	– 68% (69/101) in SSNS – 28% (19/67) in SDNS – 14% (14/103) in non-genetic SRNS – 2% (1/62) in genetic SRNS	– Prevalence of anti-nephrin increased with active disease in SSNS and SDNS – In non-genetic SRNS patients with active disease, anti-nephrin was found in 18% (13/74) patients responding to intensified immunosuppression compared to 0% (1/17) of patients with multidrug-resistant SRNS
Shirai *et al*., *KI* 2024 [7]	Signal-enhanced ELISA with commercial recombinant human nephrin ectodomain, 6xHIS-tagged, expressed in mouse myeloma cells	– 8 Japanese children with genetic FSGS – 14 Japanese children with non-genetic pFSGS and kidney transplantation, of which 11 had recurrent FSGS, 3 without recurrence – 30 controls (13 healthy, 13 MN, 4 SLE)	– 100% (11/11) in rFSGS at the time of recurrence – 33% (1/3) in non-rFSGS	
Batal *et al*., *KI* 2024 [8]	Signal-enhanced ELISA with lab-produced recombinant human nephrin ectodomain, 6xHIS-tagged, expressed in HEK293	– 38 kidney transplant patients with MCD/pFSGS, of which 21 developed recurrent MCD/pFSGS after transplantation, 17 without recurrence – Samples were collected prior to transplantation (with a median of 4 days prior to transplantation)	– 38% (8/21) in recurrent MCD/pFSGS – 0% (0/17) in non-recurrent MCD/pFSGS	– Increased risk of recurrent disease in patients positive for anti-nephrin before transplantation (HR 4.9; 95% CI 1.25–18.8; *P* < .001)

*JASN, Journal of the American Society of Nephrology; NEJM, The New England Journal of Medicine; KI, Kidney International*; HEK293, human embryonic kidney cells 293; MN, membranous nephropathy; pFSGS, primary FSGS; IgAN, IgA nephropathy; ANCA, anti-neutrophil cytoplasmic antibody; SLE, systemic lupus erythematosus; SSNS, steroid-sensitive nephrotic syndrome; SDNS, steroid-dependent nephrotic syndrome; HR, hazard ratio; CI, confidence interval.

In patients with post-transplant recurrent FSGS, the prevalence of anti-nephrin autoantibodies also varies depending on cohort characteristics and antibody detection techniques and source of recombinant protein (see below and in Table 1), ranging from 38% to 100%, and associates with shorter recurrence-free allograft survival [7, 8] (Table 1).

In an experimental animal model, active immunization of mice with recombinant murine nephrin induced anti-nephrin autoantibodies and the rapid development of a severe nephrotic syndrome with the clinical and histological phenotype of MCD [2]. Binding of anti-nephrin autoantibodies to the podocyte slit diaphragm led to an increased phosphorylation of nephrin and marked changes in the slit diaphragm proteome and the podocyte cytoskeleton. Importantly, patient-derived anti-nephrin autoantibodies were recently demonstrated to cross-react with rabbit nephrin, and their passive transfer to rabbits induced proteinuria, podocyte foot process affection and nephrin phosphorylation, providing the proof-of-concept that anti-nephrin autoantibodies are causative in the development of podocytopathic lesions [9].

## MEASUREMENT OF ANTI-NEPHRIN AUTOANTIBODIES

**Figure 1:**
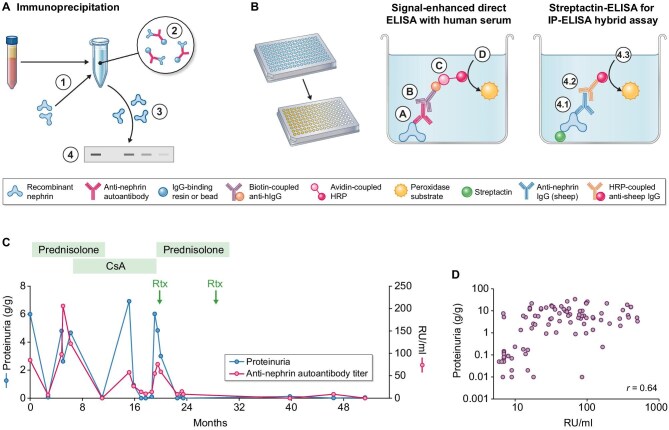
Detection of anti-nephrin autoantibodies. (**A**) Overview of immunoprecipitation for detection of anti-nephrin autoantibodies in human serum samples. (1) Human serum is incubated with recombinant nephrin to form antibody–antigen complexes. (2) Antibody-binding resin or beads are added to precipitate antibody-antigen complexes. (3) Elution of precipitated proteins releases antibodies and bound antigen. (4) Gel electrophoresis and western blotting allows for the specific detection of precipitated nephrin in samples in which anti-nephrin autoantibodies precipitated recombinant protein. (**B**) Overview of different ELISA techniques for detection of anti-nephrin autoantibodies in human serum samples. (Left) Signal-enhanced direct ELISA uses immobilized recombinant nephrin, to which human antibodies bind (A) and are detected by biotin-coupled anti-human immunoglobulin G (IgG) antibodies (B). Those are then bound by avidin-coupled horseradish peroxidase (C). Horseradish peroxidase catalyses the conversion of a chromogenic substrate indicating the amount of bound human antibodies (D). (Right) The hybrid assay of immunoprecipitation followed by ELISA quantifies the amount of previously precipitated recombinant nephrin through the binding of precipitated nephrin via its streptavidin-tag to a streptactin-ELISA plate and the specific detection of bound nephrin with a commercial sheep-derived anti-nephrin antibody (4.1) followed by the binding of a secondary anti-sheep IgG antibody coupled to horseradish peroxidase (4.2), which is quantified by chromogenic substrate (4.3). (**C**) Clinical relationships of detected circulating anti-nephrin autoantibodies in regard to disease activity and therapy response in an individual patient. (**D**) Spearman correlation of anti-nephrin autoantibody levels with proteinuria in patients with anti-nephrin-associated MCD/primary FSGS. Panels (C) and (D) reprinted with permission from Hengel *et al*., *N Engl J Med* 2024. CsA, cyclosporine A; Rtx, rituximab; RU, relative units.

The detection and quantification of circulating anti-nephrin autoantibodies has proven challenging [1, 2, 10]. Different publications report different strategies to detect anti-nephrin autoantibodies such as immunoprecipitation of recombinant human nephrin (Fig. 1A) [1, 2], signal-enhanced direct enzyme-linked immunosorbent assay (ELISA) with recombinant human nephrin (Fig. 1B, left) [1], conventional ELISA [3, 7], or a combined approach of immunoprecipitation followed by an ELISA-based quantification of precipitated recombinant nephrin as developed in our lab (see Fig. 1B, right) [2].

Immunoprecipitation is a method with several advantages over conventional antibody detection methods. (i) During the process of immunoprecipitation, patient antibodies are enriched and purified by binding to an antibody-binding resin or beads, increasing its sensitivity and allowing for the detection of low levels of circulating autoantibodies. (ii) It is highly specific as it combines the need for two independent antibody–antigen complexes to form in order to obtain a positive readout (i.e. the binding of the serum-derived autoantibody to its target antigen in solution plus the binding of the target-specific antibody to detect the precipitated antigen). However, this advantage may theroretically come at the risk of antigen detection as a consequence of antigen binding to the resin [11]. This risk requires adequate procedural controls as we demonstrated for our immunoprecipitation assay [12]. Moreover, the detection using western blotting provides the advantage of size control of the detected antigen, further improving assay specificity and reducing false-positive results. (iii) It detects delicate antigen–autoantibody interactions as the formation of antigen–autoantibody complexes takes place under native conditions, i.e. in the absence of denaturing or reducing agents (as typically used for sample preparation during western blotting) and without immobilization of the antigen on a plastic plate (as done during ELISA), both of which can lead to reduction or even loss of autoantibody binding to the epitope(s). Collectively, these technical particularities of immunoprecipitation assays offer substantial advantages in regard to the detection of autoantibodies, especially in the context of a low-level antibody disease. However, these benefits come at the cost of high sample input (such as 30 µL of serum per assay), high labour intensity and processing time, and a western blot–based, semiquantitative readout only. By contrast, a direct ELISA with recombinant nephrin being coated to a plate, to which diluted patient serum is added, has the advantage of easy applicability, scalability, low amount of input sample (usually 1–2 µL of serum per assay) and a standard curve-normalized antibody quantification. However, standard ELISA techniques did not reliably quantify anti-nephrin autoantibodies in several studies [1, 2, 13]—possibly due to their low abundance—necessitating further signal enhancement steps such as an additional signal amplification by introducing a biotin-coupled secondary antibody (purple, Fig. 1B, left) in combination with avidin-coupled horseradish peroxidase (rose and red, Fig. 1B, left). Notably, any signal enhancement can be associated with the possibility to also increase non-specific signals leading to higher false-positive results, warranting strict control conditions in such assays.

For both immunoprecipitation and ELISA, establishing advanced protocols with appropriate negative control conditions such as blood samples from healthy individuals, nephrotic patients with glomerular diseases other than primary podocytopathies and comparable serum composition, and samples without and/or substitutes for each input components are essential to assess and reduce non-specific, false-positive results [12].

The combination of immunoprecipitation followed by an ELISA-based quantification of the precipitated recombinant nephrin (Fig. 1B, right) can overcome some of the challenges of both approaches, resulting in a sensitive and specific quantitative measurement of anti-nephrin titre, while still requiring a substantial amount of hands-on work and higher patient sample input than simple ELISA techniques.

Another important variable to consider is the used recombinant nephrin protein. A recent preprint by Liu *et al*. comparing different detection methods of anti-nephrin as well as unpublished data from other laboratories suggest that the use of commercially available recombinant human nephrin, which is expressed in non-human mouse myeloma cells and most likely differs from human expressed nephrin in regard to post-translational modifications, causes high false-positive results [10, 14]. Other commercial anti-nephrin ELISAs raise questions considering their validity due to lacking information of the expression system of used recombinant protein as well as nephrin versions covering only as little as 7% of the human nephrin ectodomain [3]. In addition, high false-positive results are also reported for currently used ELISA protocols in patients with recent infections [13].

Another potential influence on test specificity can result from the use of varying protein tags, which are attached to recombinant proteins for example to allow their detection and purification. Pre-existing anti-tag antibodies in patient samples could possibly cause false-positive signals [15].

Lastly, non-pathogenic autoantibodies could exist as part of the healthy antibodyome in contrast to those with pathological effect in diseased patients, causing true positive, but non-disease correlating results [16, 17].

Another method to suggest the presence of anti-nephrin antibodies is the co-staining of IgG and nephrin in frozen kidney biopsy specimens and the assessment of co-localization of both molecules [1, 3, 7]. However, this technique does not unequivocally specify the IgG target antigen as nephrin, but rather suggests a possible antigen by co-localization.

Further possible assay methodologies to be developed include cell-based fluorescence techniques, which could detect the binding of circulating anti-nephrin autoantibodies from patient serum to full-length human nephrin expressed on the surface of cells *in vitro*. While still substantially differing from local conditions at the native slit diaphragm of the glomerular filtration barrier, this technique could offer the benefit of mimicking the binding conditions of anti-nephrin autoantibodies close to a cell membrane rather than in solution.

Taken together, a reliable, scalable and broadly available test is urgently warranted to improve the robust and reproducible detection of anti-nephrin autoantibodies on one hand and to provide potentially resulting benefits in diagnosis, prognostication and therapy guidance to affected patients in the near future on the other hand.

## ANTI-NEPHRIN AUTOANTIBODIES AS A NON-INVASIVE DIAGNOSTIC MARKER

Disease-specific biomarkers have long remained elusive in minimal change disease and FSGS. The identification of anti-nephrin autoantibodies now provides the opportunity to monitor and potentially diagnose anti-nephrin-associated disease by measuring a pathogenic circulating factor from a simple blood draw in the future. This opportunity comes with several advantages over the current gold standard of diagnosis by kidney biopsy due to its disease specificity and non-invasive character. First, it can be applied in patients with contraindications to a kidney biopsy such as anticoagulation therapy in the context of thromboembolic complications due to massive nephrotic syndrome. Second, it can help to specifically diagnose children with nephrotic syndrome who usually do not receive a kidney biopsy due to increased procedural risks and the need for periprocedural anesthesia. Third, anti-nephrin antibody detection could diagnose cases of coexisting glomerular diseases such as concomitant antibodies against PLA2R and nephrin or concomitant genetic disease, in which the histological picture of membranous nephropathy or the genetic diagnosis would overrule the diagnosis of an autoimmune form of minimal change disease. Fourth, anti-nephrin antibody measurement may be useful in cases in which a kidney biopsy is not informative anymore and proteinuria is absent, such as patients with anuric kidney failure or in cases of nephrectomy due to uncontrolled nephrotic syndrome. This could fundamentally change our approach to kidney transplantation in these patients to prevent (anti-nephrin-mediated) recurrent disease, implementing a screen for anti-nephrin antibodies and antibody-depleting therapies before transplantation as well for differential diagnosis of patients with post-transplant proteinuria. However, for anti-nephrin measurement to become such a valuable diagnostic and prognostic tool, further prospective studies and a broader availability of anti-nephrin autoantibody testing are needed.

## POTENTIAL PREDICTIVE VALUE

We could show a strong correlation of anti-nephrin autoantibody presence with proteinuria in children and adults with anti-nephrin-associated podocytopathy as well as a strong correlation of anti-nephrin autoantibody titre in adults (Spearman r = 0.64) with the extent of proteinuria across different time points in anti-nephrin-positive patients, which is illustrated on the individual patient and cohort level in Fig. 1C and D, respectively [2]. The correlation of anti-nephrin titre with disease activity gives hope for a potential predictive value of anti-nephrin antibody titre regarding further disease development. In two recent studies, anti-nephrin positivity was linked to steroid-responsive cases of childhood nephrotic syndrome, supporting the concept that anti-nephrin antibody measurement can help to predict disease course and therapy response in children [3, 4].

Further important data regarding a predictive value of anti-nephrin autoantibodies were recently provided in a retrospective study by Batal *et al*., in which 38 kidney transplant recipients with the histological diagnosis of MCD or primary FSGS were investigated before transplantation (serum sample collection with a median of 4 days prior to transplantation) [8]. Of those with recurrent disease after transplantation, 38% (8/21) were positive for anti-nephrin antibodies, while all 17 patients without posttransplant recurrence were negative for anti-nephrin. Furthermore, patients positive for anti-nephrin antibodies before kidney transplantation showed an increased risk of recurrent disease (hazard ratio 4.9; 95% confidence interval 1.25–18.8; *P* < .001), raising the question of the need to screen patients with a histological diagnosis of MCD or primary FSGS before transplantation and putative pre-emptive antibody-targeting therapy before kidney transplantation.

## PERSPECTIVE

The identification of anti-nephrin antibodies as the pathogenic factor in a subset of patients with primary podocytopathies opens a novel, pathomechanisms-based understanding and future classification of antibody-mediated podocytopathies in both adults and children, potentially voiding the current disease description based on histological pattern as in MCD or FSGS in adults or classification as ‘idiopathic’ nephrotic syndrome in children [18]. It paves the way for pathomechanisms-based diagnosis, prognostication and immunotherapeutic approaches in affected patients in the future [19–21]. Finally, it also provokes a multitude of new research questions, e.g. in regard to the emergence of these autoantibodies, their immunoglobulin G subclass and targeted epitope(s), or the ideal immunosuppressive strategy. Last but not least, a better understanding of the local action of anti-nephrin autoantibodies may lead to whole new, pathogenesis-based treatments for our patients suffering from autoimmune podocytopathies.

## Data Availability

No new data were generated or analysed in support of this research.
